# Dispersal limitation and environmental filtering effects: The taxonomic and functional beta diversity of ground beetles along the altitudinal gradient in Chinese warm‐temperature forests

**DOI:** 10.1002/ece3.11492

**Published:** 2024-06-25

**Authors:** Yagang Shen, Yi Zou, Kun Song, Xia Wan

**Affiliations:** ^1^ School of Resources and Engineering Anhui University Hefei China; ^2^ Department of Health and Environmental Sciences, School of Science Xi'an Jiaotong‐Liverpool University Suzhou China; ^3^ Zhejiang Tiantong Forest Ecosystem National Observation and Research Station, School of Ecological and Environmental Sciences East China Normal University Shanghai China

**Keywords:** assembly process, altitudinal gradient, carabids, community ecology, mountain biodiversity, warm‐temperature forests

## Abstract

Beta diversity patterns along environmental gradients and underlying mechanisms constitute key research inquiries in biogeography. However, ecological processes often also influence the functional traits of biological communities, making the assessment of functional β‐diversity crucial. Ground beetles (Coleoptera: Carabidae) are one of the most species‐rich groups in the insect community, displaying strong habitat specificity and morphological differences. In this study, we explored the patterns of taxonomic and functional beta diversity in ground beetle communities along the altitudinal gradient of warm‐temperature forests. By partitioning beta diversity into turnover and nestedness components, we evaluated their relationship with spatial distance. Our findings indicate a decline in species and functional trait similarity with increasing elevation and geographic distance. Further analysis attributed both types of beta diversity in carabids to a combination of dispersal limitation and environmental filtering, with elevation and geographic distance emerging as significant factors. Interestingly, forest‐type variations were found to have no impact on the beta diversity of these communities. Our study reveals the impact of environmental filtering and dispersal limitation on both taxonomic and functional beta‐diversity, shedding light on carabid community assembly in localized warm‐temperature forest areas in eastern China.

## Graphical abstract

In this study, we investigate the taxonomic and functional beta diversity patterns of ground beetle communities along the altitudinal gradient of warm‐temperature forests in eastern China. We get the conclusion that dispersal limitation and environmental filtering jointly drive the taxonomic and functional β‐diversity of ground beetles. This study provides insight into carabid community assembly in Chinese warm‐temperature forests.

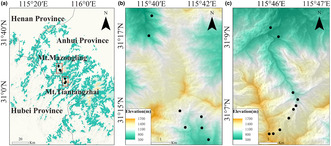

## 1 INTRODUCTION

Beta diversity has long been a focal point in ecological research, it represents the differences in composition between different communities and serves as a crucial spatial measure for understanding biodiversity patterns (Bishop et al., 2015; Soininen et al., 2018). Beta diversity resulted from two processes inlcuding species replacement and species loss and gain. The former leads to changes in unique and shared species among communities, whereas the latter not only influences this but also causes variations in species richness among them (Stachewicz et al., 2021; Wang et al., 2010), and these differences in species richness reflect the characteristic of nestedness within the communities. Decomposing beta diversity into turnover and nestedness components aids in gaining deeper insights into the spatial patterns of biological communities and their underlying driving mechanisms (Heino et al., 2019; Legendre, 2014).

Since the introduction of the concept of beta diversity, ecologists have been exploring the construction and distribution of biodiversity across geographical and environmental gradients (Legendre et al., 2005; Tuomisto & Ruokolainen, 2006) and proposed the environmental filtering hypothesis and the dispersal limitation hypothesis (Leprieur et al., 2009; Mori et al., 2015). The environmental filtering hypothesis predicts that environmental variables and species' ecological niche preferences are the main factors influencing species diversity patterns. The process of species selection along environmental gradients leads different species to appear in habitats suitable for their survival (Gutiérrez‐Cánovas et al., 2013); while the dispersal limitation recognizes spatial factors as explaining species diversity patterns (Hubbell, 2001), geographical isolation (like mountains and islands) has led to the dispersal limitation for continuously distributed species (Steinbauer et al., 2016; Zalewski et al., 2012). However, it is currently acknowledged that there is no single mechanism that can fully explain all observed patterns (Adler et al., 2007; Gaston & Chown, 2005; Leprieur et al., 2009). Hence, understanding how various ecological processes determine the patterns of beta diversity and its components among communities is crucial in biogeography studies.

Although many studies have focused on decomposing beta diversity into turnover and nestedness components (Baselga, 2010; Da Silva et al., 2018; Jiang et al., 2023), few take into account the species functional traits (Fukami et al., 2005; Siefert et al., 2013), which are crucial for revealing ecological differentiation among species (Bässler et al., 2016; Villéger et al., 2008). Two communities with different species compositions may have very similar functional traits (Loiseau et al., 2017; Villéger et al., 2013). On the other hand, when species beta diversity is high between different communities, it may not necessarily reflect higher functional beta diversity due to the similar functional traits of different species (Geldenhuys et al., 2022; Mcgill et al., 2006). Therefore, integrating species and functional beta diversity studies can provide more valuable information for understanding the distribution patterns and mechanisms of biodiversity in ecological processes (Bishop et al., 2015; Cardoso et al., 2014; Fukami et al., 2005).

Mountains have always been a focal point for the study and conservation of biodiversity (Elsen et al., 2018; Payne et al., 2020; Rahbek et al., 2019). Compared to latitudinal gradients, the environmental conditions in mountainous areas undergo significant changes over relatively short distances (Perrigo et al., 2020), which leads to complex effects of topography, climate, and landscape heterogeneity on the distribution patterns of biodiversity in mountains. Furthermore, unlike latitudinal gradients, dispersal limitations along latitude gradients may play a more significant role in shaping biodiversity patterns, whereas altitude gradients, due to their shorter spatial scale, are less affected by dispersal limitations. Considering the influence of these factors, studying the roles of dispersal limitation and environmental filtering in the distribution patterns of biodiversity in mountains can contribute to a more comprehensive understanding of the mechanisms of community assembly in mountains.

Ground beetles (Coleoptera: Carabidae) are one of the most species‐rich groups in the insect taxa (Lövei & Sunderland, 1996). Carabids are sensitive to environmental changes, showing strong habitat specificity and low interpatch dispersal rates (Wang et al., 2021; Yu et al., 2007; Zou et al., 2015). Therefore, the taxonomic and functional beta diversity of carabids might be influenced by environmental filtering and dispersal limitation. These effects on taxonomic and functional beta diversity are complex (Leão‐Pires et al., 2018; Pérez‐Sánchez et al., 2020). Specifically, environmental filtering may favor species turnover but filters for functional traits (Geldenhuys et al., 2022), species with different dispersal abilities are affected to varying degrees by spatial and environmental factors; therefore, the response of functional traits related to dispersal to spatial factors may be stronger than to environmental factors (Dalmolin et al., 2019; Heino, 2013). Despite environmental filtering and dispersal limitation always acting together on the taxonomic and functional beta diversity of biological communities, research on ground beetle diversity along both environmental and geographical gradients simultaneously remains lacking.

Located in the eastern region of China at the junction of Anhui, Hubei, and Henan provinces, the Dabie Mountains serve as the watershed between the Yangtze River and the Huai River. It belongs to the North Subtropical Zone, which is characterized by a warm and humid monsoon climate. As a representative mountainous region in eastern China, there is still a gap in our understanding of both taxonomic and functional beta diversity in carabids. Our study aims to bridge this gap by investigating the patterns of taxonomic and functional beta diversity and their respective partitioning components along an altitudinal gradient in the Dabie Mountains and to elucidate the influence of spatial and environmental factors on these patterns. Our hypotheses are as follows:

1. The process of species turnover between low and high mountains along altitudinal gradients is a well‐documented driver of altitudinal gradient patterns. Given our study area and the extent of the altitudinal range, we hypothesize that this turnover process continues to be a primary influence on the taxonomic and functional beta diversity of carabids.
2. Environmental filtering and dispersal limitation play an important and intricate role in shaping carabid beta diversity. Given the varying altitudinal distribution ranges and habitat preferences of carabids, coupled with differences in dispersal abilities, we hypothesize that environmental and spatial factors exert certain differences in their influence on the taxonomic and functional beta diversity of carabids.

## 2 MATERIALS AND METHODS

### 2.1 Study sites

**FIGURE 1:**
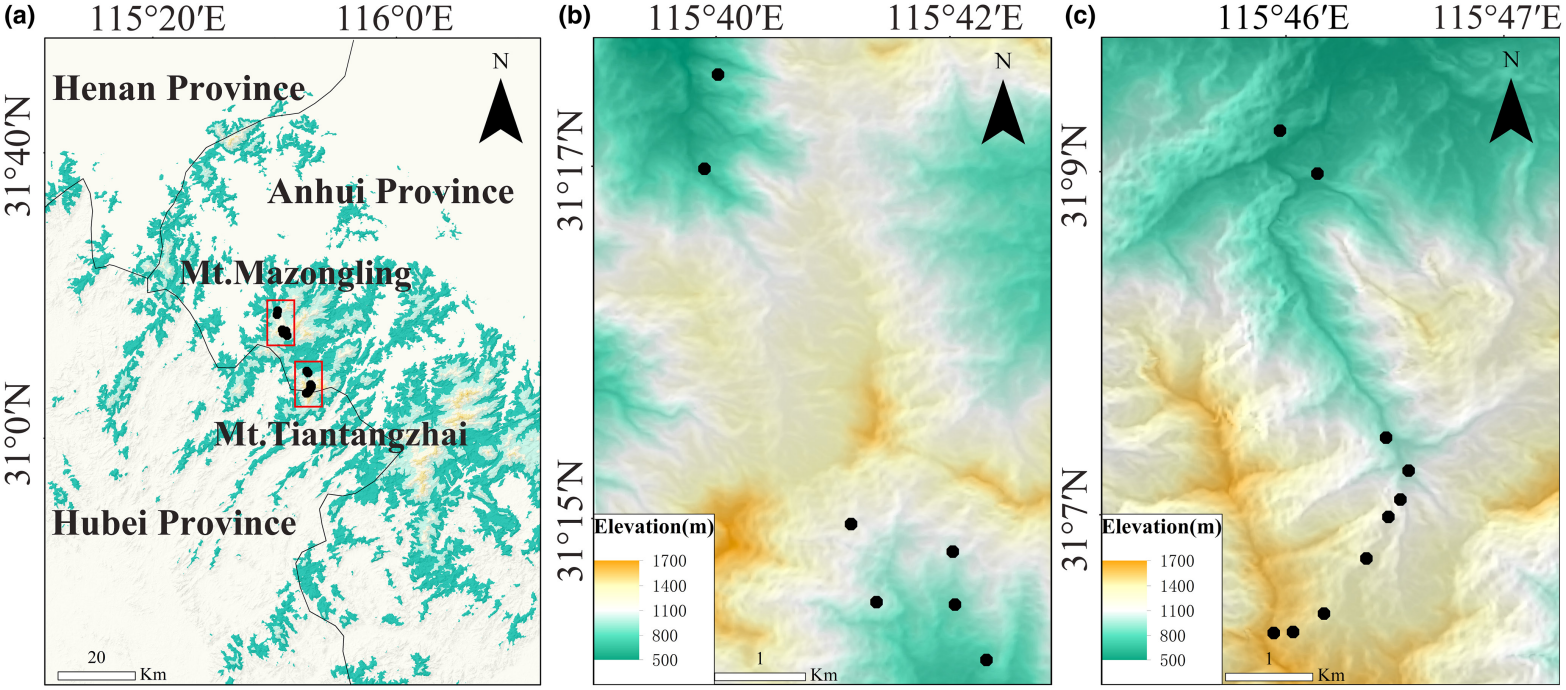
Location of the study area and sampling plots. (a) Location of two sampling sites (the shortest distance is more than 10 km), (b) plots on Mt. Mazongling and (c) plots on Mt. Tiantangzhai (the distance between any two plots is more than 150 m).

Our study area is located within the Tianma National Nature Reserve (115°20′–115°50′ E, 30°10′–31°20′ N) (Figure 1), located in the Dabie Mountains of Anhui Province. It experiences a subtropical humid monsoon climate, with an average annual temperature of 13.8°C. Extreme temperatures in July and January reach 38.1°C and −23°C, respectively. The average annual precipitation is 1489 mm, with the highest rainfall occurring from May to September (Zhang et al., 2020). The vegetation in this area vertically spans from evergreen broad‐leaved forests to deciduous broad‐leaved forests and then to coniferous forests along the altitudinal gradient. Along the elevation gradients, we established 7 and 10 plots on Mt. Mazongling (665–1270 m) and Mt. Tiantangzhai (630–1587 m), respectively. Each plot is 20 m × 20 m in size.

### 2.2 Carabids sampling

From mid‐June to mid‐October 2023, a total of 1345 ground beetles belonging to 34 species were collected from 17 plots. Each plot was subdivided into four 10 × 10 m subplots. Within each plot, five traps were placed: one at the center and one at the center of each of the four subplots. Each trap consisted of a plastic cup approximately 500 mm in volume (12.5 cm in height and 9.0 cm in diameter), with a small opening approximately 1 cm in diameter located approximately one‐third down from the top to allow overflow of rainwater. To kill and preserve specimens, traps were filled with saturated salt solution (NaCl) at approximately 50% capacity, and detergent was added to reduce surface tension. Saturated saltwater was chosen for its cost‐effectiveness and minimized the attraction of beetles and other animals directly into the traps. Although this method was used instead of lure‐based sampling, it still yielded a substantial capture rate (Kotze et al., 2011; Zou et al., 2014). All traps at the sampling points were emptied every 2 weeks, and the collected samples were identified as morphospecies. We pooled all samples collected during the entire sampling period for subsequent analysis, and all samples were analyzed at the plot level. A full set of species is stored at the Chinese Academy of Sciences and Anhui University.

### 2.3 Functional traits

For functional traits, we selected eight morphological traits: body length, head length, head width, antenna length, pronotum length, pronotum width, elytra length, and abdomen width (details information selected see Appendix S1: Table S3). For species with sample sizes exceeding 10 individuals, 10 individuals were selected for measurement, and their averages were calculated. For species with fewer than 10 individuals, all individuals were measured to calculate the average. As morphological feature measurements are often highly correlated with body size (Hagge et al., 2021), all functional traits were standardized by body length. These traits were labeled as relative traits, where each logarithmically transformed trait value was regressed against the logarithmically transformed body length (Barton et al., 2011). Subsequently, residuals were extracted from the fitted model as our functional trait values, representing the deviation of specific species' traits from the expected values given their body length.

### 2.4 Spatial and environmental factors

We recorded all tree species (diameter at breast height [DBH] > 5 cm were recorded) and their abundance in each plot as representatives. Subsequently, we documented the average temperature, humidity, slope, canopy cover, and mean diameter at breast height for each plot throughout the sampling period. The average air temperature and humidity for the entire sampling period (from June 15 to October 15, 2023) were used. Leaf litter depth was measured at a random point 1 m beside each trap and averaged. Subsequently, utilizing geographical coordinates, we obtained open‐access satellite images from the European Sentinel‐2A satellite covering the entire study area on September 1, 2023. Using ENVI 5.4 software, we extracted the average normalized difference vegetation index (NDVI) values within a radius of 100 m around each geographical coordinate. Latitude and longitude were considered spatial factors, whereas other factors were considered environmental factors (see Humphrey et al., 1999; Huo et al., 2014; Wang et al., 2021 for details information on environmental factors measured).

### 2.5 Statistical analyses

Based on the presence/absence data of species, the Sørensen dissimilarity index and its functional analogs were utilized to compute the taxonomic and functional β‐diversity among communities. We decomposed the two β‐diversity diversity measures into their turnover and nestedness components to assess the contributions of different components to β‐diversity (Baselga, 2010; Villéger et al., 2008). Before calculating functional β‐diversity, we calculated the functional space using the mFD package based on PCA (Magneville et al., 2022) and conducted relevant assessments (see Appendix S1), and the first four axes were selected as input data for computing the functional trait β‐diversity.

The relationships between β‐diversity and its components with elevation and geographical distance were tested using Mantel and partial Mantel tests. Geographical distances were calculated from latitude and longitude using the geosphere package and after an ln(x + 1) transformation.

Finally, we utilized multiple regression on distance matrices (MRM) to analyze the impact of spatial and environmental factors on β‐diversity and its components. Before this analysis, we calculated the correlations between environmental factors, which revealed a high correlation between temperature and elevation; consequently, the temperature factor was excluded from the analysis. To represent differences in tree composition, Bray‐Curtis distances were computed based on tree abundance data. The Euclidean distance matrix for other environmental factors was derived using the vegdist function, and all distance matrices were standardized before performing the MRM analysis.

We used the packages “FD” and “betapart” to calculate and partition taxonomic and functional beta diversity (Baselga et al., 2023; Laliberté & Legendre, 2010). We used the “MuMIn” package to calculate the MRM models (Bartoń, 2020). We used the “Vegan” package to calculate the distance matrices and perform Mantel and partial Mantel tests (Oksanen et al., 2022). We used the “Hmisc” package to calculate the correlation of environmental factors (Harrell Jr, 2023). All steps run in the R 4.3.0 version (R Core Team, 2023).

## 3 RESULTS

### 3.1 Beta diversity and its partitioning components

**FIGURE 2:**
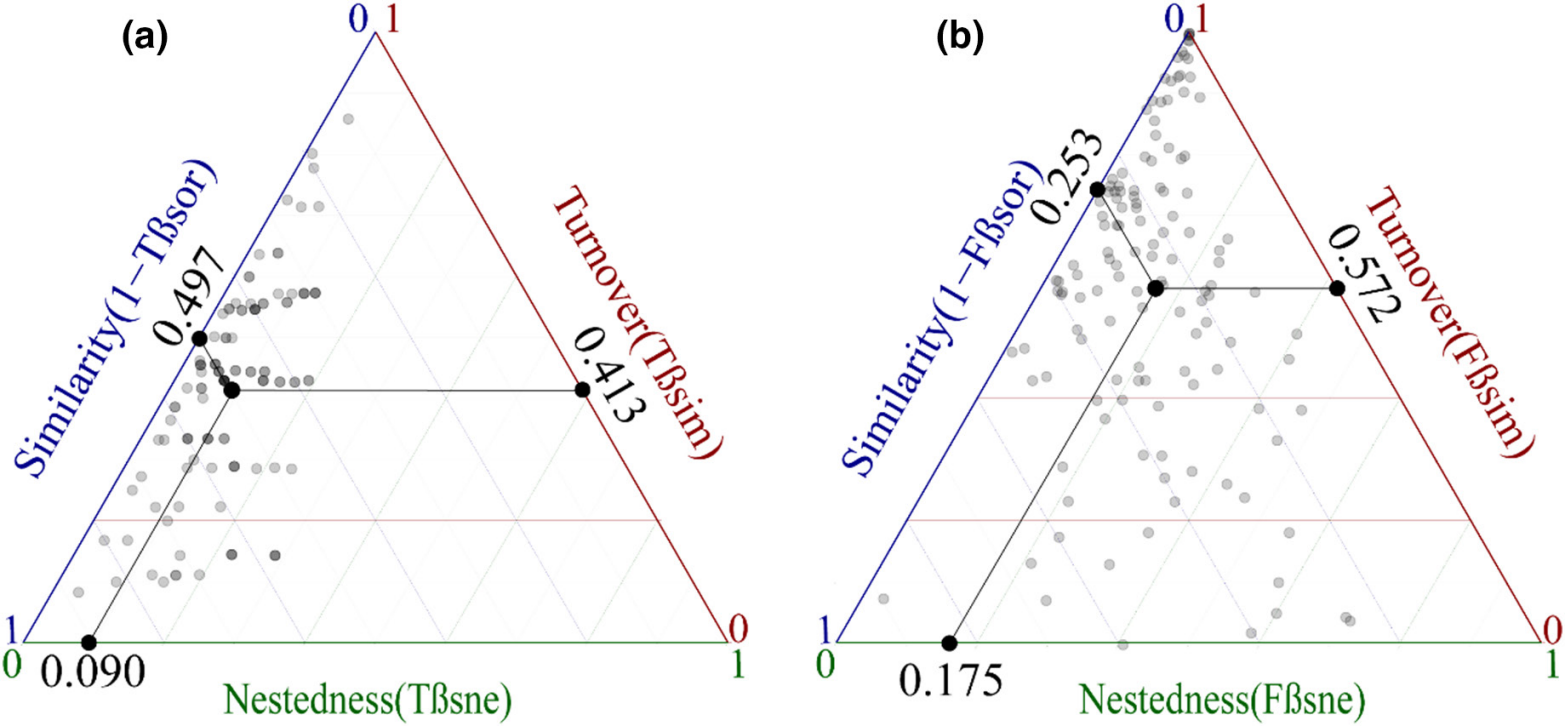
Taxonomic and functional β‐diversity and its components (turnover and nestedness). (a) Taxonomic β‐diversity, (b) Functional β‐diversity. The large black points represent the mean values of turnover (Tβsim, Fβsim), nestedness (Tβsne, Fβsne), and similarity (1‐Tβsor, 1‐Fβsor).

The average values of taxonomic β‐diversity and its turnover and nested components are 0.503, 0.413, and 0.090, respectively, whereas the average values of functional β‐diversity and its turnover and nestedness are 0.747, 0.572, and 0.175, respectively (Figure 2). The total dissimilarity in functional traits is greater than in taxonomic, and both taxonomic and functional beta diversity are dominated by their respective turnover components, the contribution of nested components to functional beta diversity (23.43%) was significantly higher than that of taxonomic beta diversity (17.78%).

### 3.2 Influence of elevational and spatial distances on beta diversity

**FIGURE 3:**
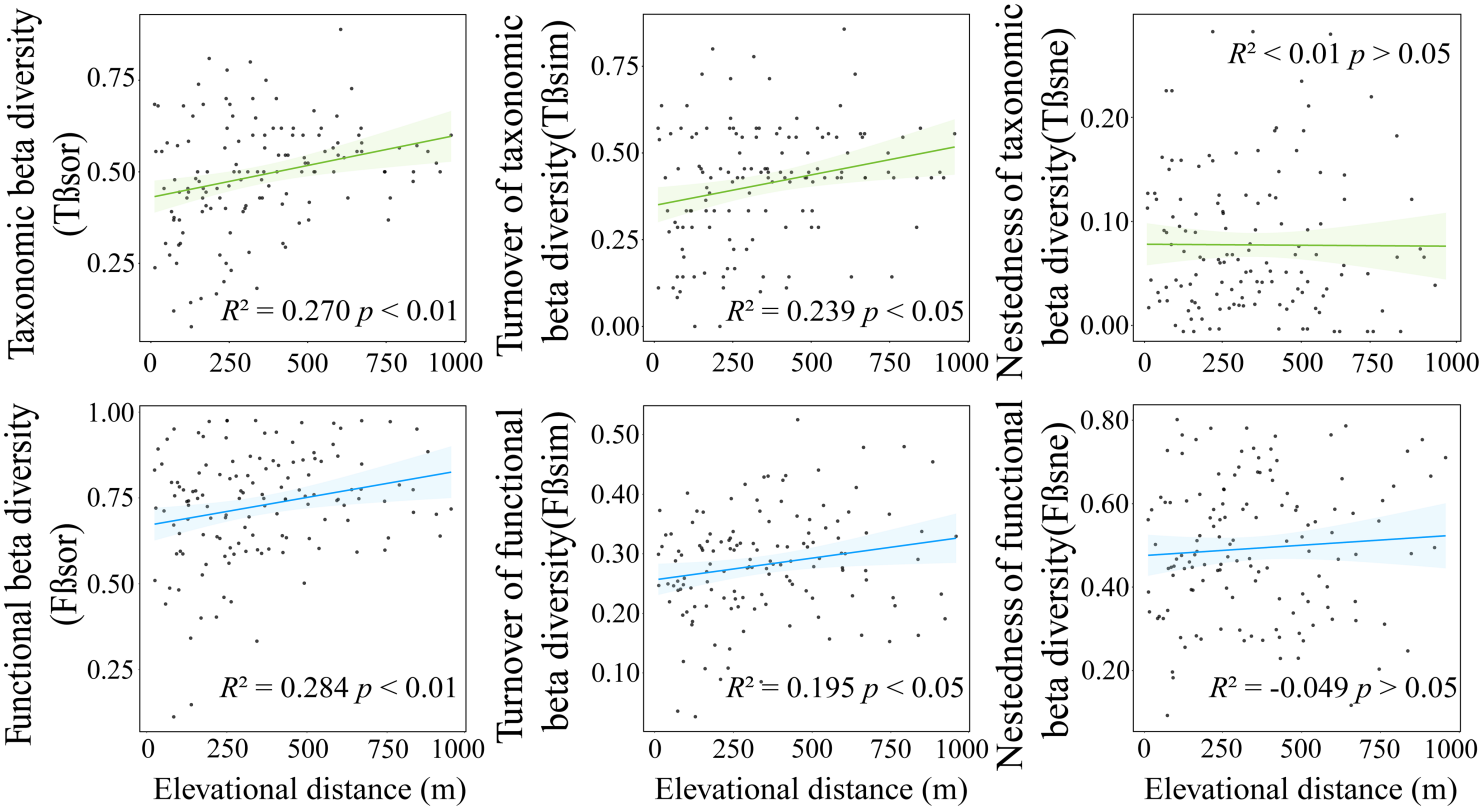
The relationship of taxonomic and functional β‐diversity and its components (turnover and nestedness) with difference elevational distance (Mantel tests).

**FIGURE 4:**
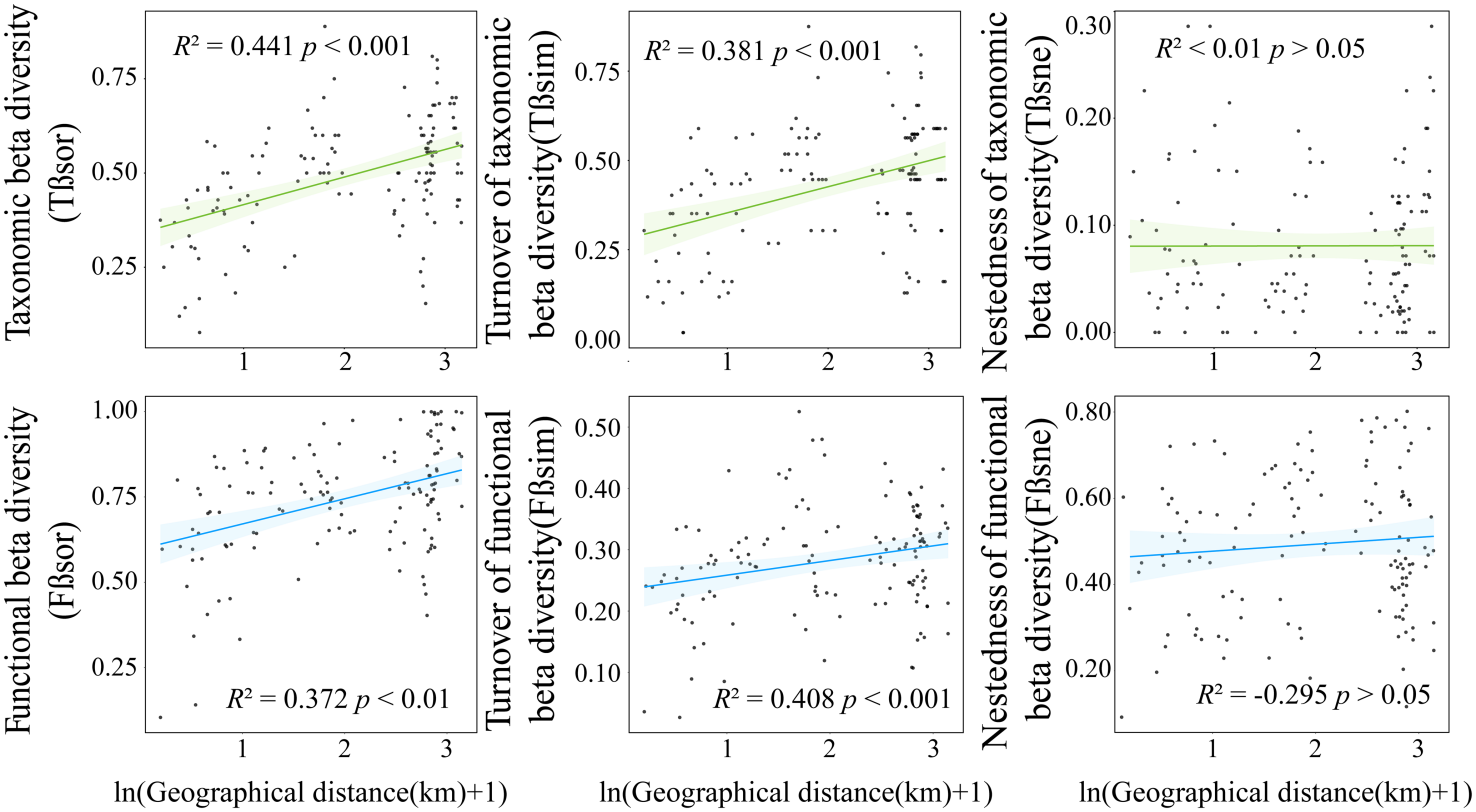
The relationship of taxonomic and functional β‐diversity and its components (turnover and nestedness) with difference geographical distance (partial Mantel tests).

Both elevational and spatial distances exhibit a significantly positive correlation with the taxonomic and functional β‐diversity of the ground beetle community in the study area (Figures 3 and 4). As altitude and spatial distance increase, the composition of species and functional traits among communities changes, resulting in greater differences between communities and consequently higher β‐diversity. Upon decomposition of beta diversity into the turnover and nestedness components, it was found that elevational and spatial distance affects only the turnover of species between communities, showing no significant influence on the nestedness. Furthermore, the response of the turnover component of β‐diversity to increasing altitude and spatial distance remains consistent with the overall response of β‐diversity. Changes in altitude and increased spatial distance primarily affect the β‐diversity of the ground beetle community through the turnover of species and the composition of functional traits between communities.

### 3.3 Contributions of spatial and environmental factors

Through the multiple regression on distance matrices (MRM) model, the relative contributions of spatial and environmental factors to the taxonomic and functional β‐diversity and their respective components were assessed (Table 1). For taxonomic β‐diversity, geographical distance, elevation, slope, and DBH together accounted for 27.8% and 28.3% of turnover and nestedness components, and NDVI explains 8.3% of the nested component. For functional beta diversity, our model indicates that geographical distance, slope, and NDVI collectively explain 21.8% of the functional beta diversity. Regarding the turnover component of functional beta diversity, only elevation alone accounts for 3.4% of the explanation. Moreover, none of the factors in the full model were found to be significant in explaining the nestedness component of functional beta diversity (*p* > .05).

**TABLE 1:** Model coefficients, *R* ^2^ and *p* values of the multiple regression on beta diversity and its components for models with explanatory dissimilarity matrices.

	Explanatory dissimilarity matrices									Full model	
	GeoDis	EleDis	AH	Slope	TC	DBH	LD	NDVI	Canopy	*R* ^2^	*p*
Taxonomic β‐diversity	0.116**	0.106**	NS	0.018*	NS	0.015*	NS	NS	NS	.278	.003
Turnover (Tβsim)	0.187**	0.163**	NS	0.026*	NS	0.020*	NS	NS	NS	.283	.003
Nestedness (Tβsne)	NS	NS	NS	NS	NS	NS	NS	0.048*	NS	.083	.288
Functional β‐diversity	0.011*	NS	NS	0.009*	NS	NS	NS	0.011*	NS	.218	.005
Turnover (Fβsim)	NS	0.034**	NS	NS	NS	NS	NS	NS	NS	.128	.002
Nestedness (Fβsne)	NS	NS	NS	NS	NS	NS	NS	NS	NS	.097	.309

*Note*: *p < .05; **p < .01.

Abbreviations: AH, air humidity; Canopy, canopy cover; DBH, diameter at breast height; EleDis, elevational distance; GeoDis, geographical distance; LD, litter depth; NS, nonsignificant; TC, tree composition.

## 4 DISCUSSION

Our study investigated the altitudinal patterns and driving mechanisms of both the taxonomic and functional β‐diversity of ground beetles in forests in eastern China. The patterns observed in both taxonomic and functional β‐diversity were primarily attributed to the turnover of species and functional traits between communities. Our research findings indicate that dispersal limitation and environmental filtering commonly influence the β‐diversity of both taxonomic and functional aspects of ground beetles. Additionally, environmental factors have different effects on the taxonomic and functional beta diversity. It was observed that the spatial factors (geographical distance and elevation) had the most prominent and dominant effect on these diversity patterns.

### 4.1 Beta diversity and its partitioning components

Functional beta diversity (0.747) was higher than species beta diversity (0.503) among communities (Figure 2), indicating lower dissimilarity in the species composition compared to the functional composition of carabid communities in the study area. Both species and functional beta diversity were primarily driven by high turnover components, suggesting that the beta diversity of carabids in the study area mainly results from the turnover of species and functional traits across space or between communities, a finding consistent with other related research results (Da Silva et al., 2018; Fontana et al., 2020; Soininen et al., 2018).

The turnover component's component's contribution to species beta diversity (82.22%) was higher than that to functional beta diversity (76.57%), indicating that carabids exhibit a much higher turnover rate in terms of community or spatial replacement than in the turnover of their functional traits. When considering the overall beta diversity difference (0.503 vs. 0.747), may the occurrence of rare or habitat‐preference species during community or spatial turnover leads to significant differences in the community's functional structure (Burner et al., 2022; Tang et al., 2006).And o Our results show the higher contribution of the functional beta diversity's nested component (27.89% vs. 17.78%).

### 4.2 Responses to spatial and elevational distance

Although there are limited studies on altitudinal gradient patterns of beta diversity in ground beetles, taxonomic beta diversity and turnover have been found to increase with altitude, which aligns with previous related studies (Carvalho et al., 2023; Zou et al., 2015), our results demonstrate a strong influence of altitude variation on both taxonomic and functional beta diversity and their turnover components (Figure 3). Furthermore, despite the close geographical distance between Mt.Mazongling and Mt.Tiantangzhai (approximately 10 km apart), this limited geographic distance plays an equally significant role in the differentiation of community structure and function. On the one hand, we speculate that this influence could depend on the limited dispersal ability of ground beetles. On the other hand, the landscapes between Mt.Mazongling and Tiantangzhai consist largely of farmlands and villages, and previous research has demonstrated the impact of ecosystems such as grasslands and farmlands on ground beetles (Fusser et al., 2017; Philpott et al., 2019). Habitat patches formed by these landscapes can increase beta diversity turnover (Yan et al., 2021) while limiting species dispersal (Ewers & Didham, 2007). Therefore, carabids rely on these landscapes to carry out significant turnover processes among communities. Spatial factors have long been recognized as crucial drivers shaping the distribution patterns of beta diversity (Brown et al., 2018), and our findings underscore their considerable influence on a local scale.

### 4.3 Environmental and spatial effects on beta diversity

Heterogeneity in certain environmental factors within habitats leads to environmental filtering, where niche processes play a crucial role (Jiang et al., 2023; Soininen et al., 2018). Our results demonstrate the importance of environmental filtering in shaping the pattern of beta diversity, while the dispersal limitation caused by geographical distance also had a significant impact (Table 1). Slope, as a topographical factor, was found to influence the turnover of species in the classification beta diversity (Huo et al., 2014; Yu et al., 2015).Meanwhile, tree DBH is generally considered an indicator of forest maturity, and it has a positive impact on the species composition of beetle communities (Zou et al., 2019). However, the impact of environmental factors on species beta diversity in these habitats is far less than that of geographical distance and elevation.

Geographical distance, slope, and NDVI explain a certain amount of functional β‐diversity (Table 1), whereas its turnover component is predominantly influenced by elevation factors. Considering that our functional traits were selected based on morphology, this is a reasonable result, as morphological functional traits (such as body size) can affect carabid stride length and walking speed (Barton et al., 2011; Krasnov et al., 1996; Seidler & Plotkin, 2006), thus carabids are influenced by spatial distance and slope during dispersal. Traits, such as antenna length affect microhabitat preference (Barton et al., 2011; Fountain‐Jones et al., 2015), and NDVI, as a representative of ground vegetation, may exert selective pressure on carabids in this regard (Wang et al., 2021). The different responses of functional beta diversity and its turnover component to spatial and environmental factors indicate that other underlying factors drive the functional beta diversity and its components of carabids. Furthermore, future research should take into account additional types of functional traits beyond morphology.

Furthermore, forest ecosystems serve as crucial habitats for ground beetles, and different types of forest and plant compositions can affect the distribution of beetles by influencing microhabitats on the ground. However, we found that differences in community beta diversity were not sensitive to forest types (tree composition). Previous studies have commonly suggested a strong correlation between forest structure complexity and ground beetle community patterns (Axmacher et al., 2023; Zou et al., 2014, 2015). Previous studies highlighted the scale‐dependent influence of dispersal limitation (Yao et al., 2023), therefore, within the scope of our smaller scale study, differences in forest structure are not the primary cause of differences in carabid communities, particularly since the majority of the forests in our study area are mature natural forests (Zou et al., 2019), compared to woody plants, herbaceous plants or shrubs that influence the ground microhabitat may have a more significant impact on the community structure of carabids. Overall, our results indicate that spatial (geographic) distance and elevation play a primary role in taxonomic and functional beta diversity, especially with respect to species beta diversity. Other environmental factors have limited effects on beta diversity on the scale of our study, with dispersal limitation playing a larger role compared to environmental filtering.

## 5 CONCLUSION

Our study provides important information on altitudinal patterns and the influencing mechanisms of biodiversity in the subtropical forests of low‐altitude mountain ranges in eastern China. The high levels of beta diversity exhibited in the classification and functionality of beetle communities in this area highlight their significant conservation value in terms of biodiversity. However, the impact of environmental factors on both taxonomic and functional beta diversity is limited and not necessarily consistent, they may be influenced by different environmental factors and we need highlights the need for multifaceted research on beta diversity to better understand the influence of the environment on biological communities. This underscores the necessity to investigate various aspects of diversity to fully understand ecological processes. Future research efforts should be directed toward larger scales, encompassing a more extensive array of taxa and functional combinations, to better understand the dynamic alterations in biological communities at broader scales and their responses to geographical environments. Such studies would offer deeper insight into biodiversity conservation and ecosystem management.

## AUTHOR CONTRIBUTIONS

**Yagang Shen:** Conceptualization (equal); data curation (equal); formal analysis (lead); investigation (lead); writing – original draft (lead). **Yi Zou:** Data curation (equal); investigation (equal); methodology (equal); supervision (equal); writing – review and editing (lead). **Kun Song:** Funding acquisition (equal); investigation (equal); supervision (equal); writing – review and editing (equal). **Xia Wan:** Funding acquisition (equal); investigation (equal); project administration (lead); supervision (lead); visualization (equal); writing – review and editing (lead).

## CONFLICT OF INTEREST STATEMENT

The authors declare no potential conflict of interest.

## Supporting information


Appendix S1

## Data Availability

The data that support the findings of this study are available in Appendix S1 of this article.
