# Supplementary material for: Dispersal limitation and environmental filtering effects: The taxonomic and functional beta diversity of ground beetles along the altitudinal gradient in Chinese warm‐temperature forests
Source: Ecol Evol. 2024 Jun 25;14(6):e11492. doi: 10.1002/ece3.11492 (PMC11199336; doi:10.1002/ece3.11492)
Supplement: Supplementary file 1 — Appendix S1 [file ECE3-14-e11492-s001.docx]

**SUPPORTING INFORMATION**

**TABLE S1**. Locations, elevation and forest types of sampling plots.

|  | Plot ID | | Elevation | Longitude | Latitude | Forest type |
| --- | --- | --- | --- | --- | --- | --- |
| Mt.Mazongling | | MZL1 | 665 | 115.684 | 31.306 | Evergreen deciduous broad-leaved forest |
|  |  | MZL2 | 750 | 115.682 | 31.297 | Mixed coniferous and broad-leaved forest |
|  |  | MZL3 | 841 | 115.710 | 31.248 | Mixed coniferous and broad-leaved forest |
|  |  | MZL4 | 930 | 115.707 | 31.254 | Mixed broad-leaved forest |
|  |  | MZL5 | 1032 | 115.707 | 31.259 | Mixed broad-leaved forest |
|  |  | MZL6 | 1150 | 115.699 | 31.254 | Mixed broad-leaved forest |
|  |  | MZL7 | 1270 | 115.697 | 31.262 | Mixed coniferous and broad-leaved forest |
| Mt.Tiantangzhai | | TTZ1 | 630 | 115.765 | 31.166 | Deciduous broad-leaved forest |
|  |  | TTZ2 | 677 | 115.769 | 31.161 | Deciduous broad-leaved forest |
|  |  | TTZ3 | 917 | 115.776 | 31.134 | Evergreen deciduous broad-leaved forest |
|  |  | TTZ4 | 990 | 115.778 | 31.131 | Evergreen deciduous broad-leaved forest |
|  |  | TTZ5 | 1084 | 115.777 | 31.128 | Evergreen deciduous broad-leaved forest |
|  |  | TTZ6 | 1166 | 115.776 | 31.126 | Evergreen deciduous broad-leaved forest |
|  |  | TTZ7 | 1293 | 115.774 | 31.122 | Deciduous broad-leaved forest |
|  |  | TTZ8 | 1422 | 115.769 | 31.116 | Mixed coniferous and broad-leaved forest |
|  |  | TTZ9 | 1513 | 115.766 | 31.114 | Mixed coniferous and broad-leaved forest |
|  |  | TTZ10 | 1587 | 115.764 | 31.114 | Coniferous forest |

**TABLE S2**. Species identified of ground beetles sampled in each plot.

| Species | Plots | | | | | | | | | | | | | | | | |
| --- | --- | --- | --- | --- | --- | --- | --- | --- | --- | --- | --- | --- | --- | --- | --- | --- | --- |
|  | Mt.Mazongling | | | | | | | Mt.Tiantangzhai | | | | | | | | | |
|  | 1 | 2 | 3 | 4 | 5 | 6 | 7 | 1 | 2 | 3 | 4 | 5 | 6 | 7 | 8 | 9 | 10 |
| *Amara goniodera* |  |  |  |  |  |  | √ |  |  |  |  |  |  |  |  |  |  |
| *Amara simplicidens* |  |  |  |  | √ |  |  |  |  |  |  |  |  |  |  |  |  |
| *Archipatrobus deuvei* |  |  |  |  |  |  | √ |  |  |  |  |  |  |  |  |  |  |
| *Carabus protenes* | √ | √ | √ |  |  |  |  | √ | √ |  |  |  | √ | √ | √ |  | √ |
| *Carabus lafossei* |  | √ |  |  |  |  |  | √ | √ |  |  |  |  |  |  | √ |  |
| *Carabus casaleianus* |  |  | √ |  |  |  |  |  |  |  |  |  |  |  |  |  |  |
| *Chlaenius bimaculatus* | √ | √ | √ | √ | √ |  |  | √ | √ |  |  | √ | √ | √ | √ | √ | √ |
| *Chlaenius tetragonoderus* | √ |  | √ |  |  |  |  |  |  |  |  |  |  |  |  |  |  |
| *Diplocheila zeelandica* |  |  | √ |  |  |  |  |  |  |  |  |  |  |  |  |  |  |
| *Dolichus davidis* |  |  |  |  |  |  | √ |  |  |  |  |  |  |  |  |  |  |
| *Eucolpodes japonicum chinadense* |  |  |  |  |  |  |  |  |  |  |  |  |  |  |  |  | √ |
| *Harpalus* sp. |  | √ |  |  | √ |  | √ |  |  |  |  |  |  |  |  |  |  |
| *Nebria chinensis* |  |  |  |  |  |  | √ |  |  |  |  |  | √ | √ |  |  |  |
| *Platynus protensus* |  |  |  |  |  |  |  |  |  |  |  |  | √ |  |  |  | √ |
| *Pristosia chinensis* |  |  |  |  |  | √ |  |  | √ | √ | √ |  |  |  |  |  |  |
| *Pristosia nitidula* |  |  |  |  |  | √ |  |  |  |  |  |  |  |  |  |  |  |
| *Pristosia* sp |  |  |  |  |  |  |  |  | √ | √ | √ | √ | √ | √ | √ |  |  |
| *Pristosia suensoni* |  |  |  |  |  |  |  |  |  |  |  |  | √ |  |  |  |  |
| *Pseudoodes*sp |  |  |  |  |  |  |  | √ |  |  |  |  |  |  |  |  |  |
| *Pterositchus* sp.1 | √ |  | √ | √ | √ | √ | √ |  |  | √ | √ | √ | √ | √ | √ | √ | √ |
| *Pterostichus* sp. 2 |  |  |  |  |  | √ |  |  |  |  | √ | √ | √ | √ |  |  | √ |
| *Pterostichus* sp. 3 | √ |  |  |  |  | √ |  |  |  |  |  | √ |  | √ | √ |  |  |
| *Pterostichus pratti* | √ | √ | √ | √ |  | √ |  | √ | √ | √ | √ | √ | √ | √ | √ | √ | √ |
| *Pterostichus kiangsu* | √ |  |  | √ |  | √ |  | √ |  | √ | √ | √ | √ | √ | √ |  |  |
| *Pterostichus crassiapex* |  |  |  |  |  |  |  |  |  |  |  |  |  | √ | √ |  |  |
| *Synuchus chinensis* |  | √ | √ | √ | √ | √ | √ | √ | √ | √ | √ |  |  |  |  |  |  |
| *Synuchus nitidus reticulatus* |  | √ | √ | √ | √ | √ | √ |  | √ | √ | √ | √ | √ | √ | √ | √ | √ |
| *Synuchus* sp.1 |  |  |  |  |  |  |  |  |  |  | √ | √ | √ | √ | √ | √ |  |
| *Synuchus* sp.2 |  |  |  |  |  |  | √ |  |  | √ |  |  | √ | √ | √ |  |  |
| *Synuchus* sp.3 |  |  |  | √ |  | √ |  |  | √ | √ | √ |  |  |  |  |  |  |
| *Synuchus* sp.4 |  |  | √ | √ | √ | √ | √ | √ | √ | √ | √ |  |  |  |  |  |  |
| *Synuchus* sp.5 |  |  | √ | √ | √ | √ | √ | √ | √ | √ | √ |  |  |  |  |  |  |
| *Trigonognatha* sp |  |  |  |  | √ | √ |  | √ | √ | √ | √ | √ | √ | √ | √ | √ | √ |
| *Trigonotoma lewisii* |  |  |  |  |  |  |  | √ |  |  |  |  |  |  |  |  |  |

√ indicated that the species occurred in the plot.

**TABLE S3.** Lists of morphological traits measured and their ecological functions

| Functional Traits | Trait description | Ecological function |
| --- | --- | --- |
| Body length | Total length of beetle, excluding appendages or setae | fecundity; foraging behavior; dispersal ability(Barton et al., 2011; Fountain-Jones et al., n.d.; Gillespie et al., 2017; Pérez-Sánchez et al., 2020) |
| Head length | Maximum dorsal length of head | resource use(Thompson, 1992) |
| Head width | Maximum dorsal width of head | microhabitat use; resource use (Barton et al., 2011; Thompson, 1992) |
| Pronotum length | Maximum dorsal length of pronotum along medial line | protective trait; foraging behavior(Barton et al., 2011; Fountain-Jones et al., n.d.) |
| Pronotum width | Maximum dorsal width of pronotum | microhabitat use (Barton et al., 2011) |
| Antenna length | Maximum length of an antenna, including all antennomeres | habitat preference (Barton et al., 2011; Fountain‐Jones et al., 2015; Vandewalle et al., 2010) |
| Elytra length | Maximum dorsal length of elytra along medial line | wing development; dispersal ability (Barton et al., 2011; Ribera et al., 1999a;) |
| Abdomen width | Maximum dorsal width of abdomen | microhabitat use (Barton et al., 2011) |

Note: traits measurement method and selection from Barton et al. (2011); Hagge et al. (2021) and Pérez-Sánchez et al., (2020).

**TABLE S4.** The first four axes from the principal coordinate analysis (PCA) of relative functional traits.

| Species | PC1 | PC2 | PC3 | PC4 |
| --- | --- | --- | --- | --- |
| *Amara goniodera* | 0.3019 | 2.7317 | -0.3077 | 0.1221 |
| *Amara simplicidens* | 1.5771 | 0.8684 | -0.9724 | -2.1559 |
| *Archipatrobus deuvei* | 0.1267 | -1.229 | 0.2689 | 0.9055 |
| *Carabus protenes* | -2.2593 | 0.5904 | 1.5030 | -0.0071 |
| *Carabus lafossei* | 0.3228 | -1.1030 | 3.0091 | -0.1181 |
| *Carabus casaleianus* | -1.3177 | 0.4838 | 2.3536 | 0.0037 |
| *Chlaenius bimaculatus* | -1.2908 | 1.0935 | 1.7322 | 0.4644 |
| *Chlaenius tetragonoderus* | -0.4745 | 0.5931 | -0.1328 | 0.0954 |
| *Diplocheila zeelandica* | -0.6739 | 0.6521 | -0.1754 | 0.0452 |
| *Dolichus davidis* | -1.7879 | -0.7102 | -0.3533 | -1.0639 |
| *Eucolpodes japonicum chinadense* | -3.1695 | -1.2147 | -0.3304 | -1.2460 |
| *Harpalus* sp | 0.3875 | 2.3967 | -0.4709 | 0.0075 |
| *Nebria chinensis* | -1.5250 | 0.2883 | 0.0413 | -0.2618 |
| *Platynus protensus* | -2.5301 | -1.5936 | -0.2518 | -1.8667 |
| *Pristosia chinensis* | -0.2765 | -0.6904 | 0.0903 | 0.5419 |
| *Pristosia nitidula* | 3.6388 | -2.0504 | 0.3845 | -2.4019 |
| *Pristosia* sp | 0.2163 | -0.6405 | 0.2045 | 1.1589 |
| *Pristosia suensoni* | -0.0043 | -0.9962 | -0.0290 | 0.6989 |
| *Pseudoodes*sp | -2.2345 | 2.3305 | -1.0312 | -1.2070 |
| *Pterositchus* sp.1 | 1.8959 | -0.8767 | -1.1999 | 0.3258 |
| *Pterostichus* sp. 2 | 0.7066 | 1.1538 | -0.7496 | 0.1016 |
| *Pterostichus* sp. 3 | 1.1587 | -1.0137 | -0.5939 | 0.3237 |
| *Pterostichus pratti* | 1.5553 | 0.7088 | 1.0299 | -0.4061 |
| *Pterostichus kiangsu* | 1.1408 | 0.7444 | -0.5939 | 1.0387 |
| *Pterostichus crassiapex* | 0.8447 | 3.3178 | 0.1858 | 1.5126 |
| *Synuchus chinensis* | -0.4442 | -0.6576 | -1.5554 | -0.0346 |
| *Synuchus nitidus reticulatus* | 0.2930 | -0.3541 | 0.2138 | 0.4355 |
| *Synuchus* sp.1 | 0.5592 | -1.8514 | 0.0903 | 1.0664 |
| *Synuchus* sp.2 | 0.0514 | -2.8360 | -0.1328 | 0.9818 |
| *Synuchus* sp.3 | -0.9926 | 0.3058 | -0.9724 | 0.1429 |
| *Synuchus* sp.4 | -1.0679 | 0.6469 | -1.0913 | 0.6600 |
| *Synuchus* sp.5 | -0.3129 | -2.4774 | -1.0611 | 1.3725 |
| *Trigonognatha* sp | 2.7575 | 0.7979 | 0.1000 | -0.0615 |
| *Trigonotoma lewisii* | 2.8274 | 0.5912 | 0.7977 | -1.1743 |

**TABLE S5.** The eigenvalues and the proportion of variance explained by the first four axes of PCA

| Axies | Eigenvalue | Percentage of variance | Cumulative percentage of variance |
| --- | --- | --- | --- |
| PC1 | 2.3721 | 30.55 | 30.55 |
| PC2 | 2.0652 | 26.60 | 57.15 |
| PC3 | 0.9706 | 12.50 | 69.65 |
| PC4 | 0.9141 | 11.77 | 81.42 |


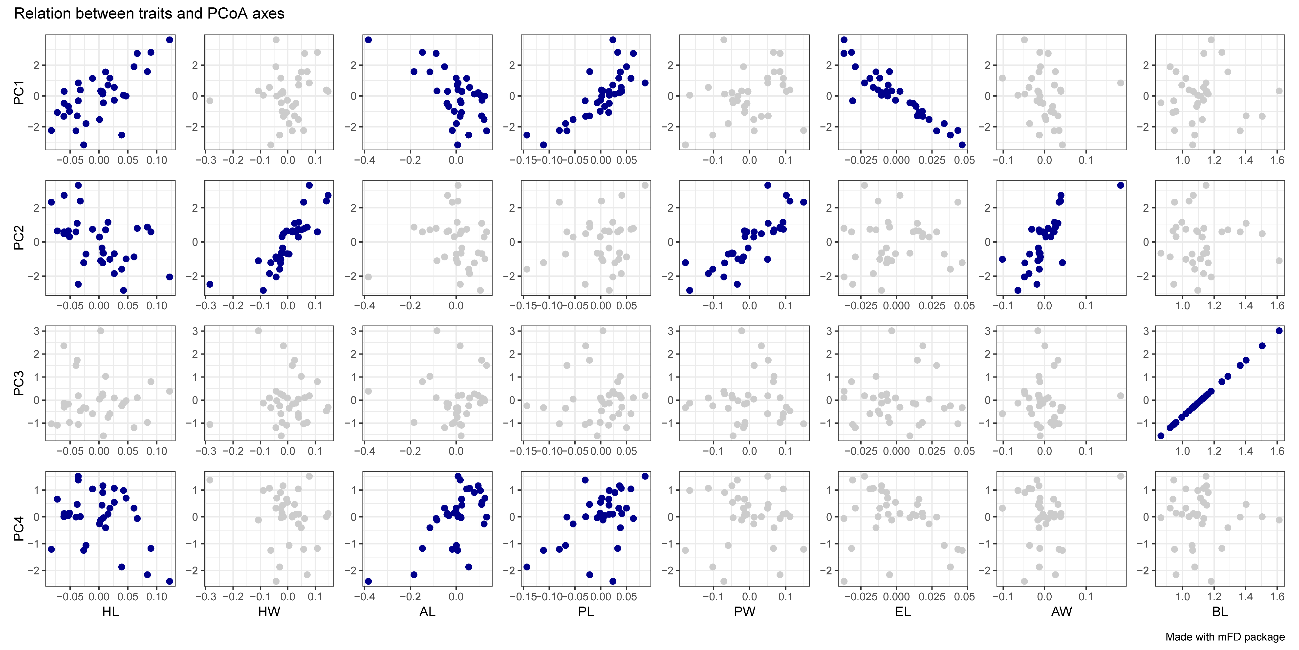
**TABLE S6.** Correlation between Traits and Axes of PCA

| Trait | Axis | Test | Value | P.value |
| --- | --- | --- | --- | --- |
| Head length | PC1 | Linear Model | 0.459 | 0.0000 |
| Head length | PC2 | Linear Model | 0.205 | 0.0072 |
| Head length | PC4 | Linear Model | 0.118 | 0.0468 |
| Head width | PC2 | Linear Model | 0.669 | 0.0000 |
| Antenna length | PC1 | Linear Model | 0.390 | 0.0001 |
| Antenna length | PC4 | Linear Model | 0.393 | 0.0001 |
| Pronotum length | PC1 | Linear Model | 0.613 | 0.0000 |
| Pronotum length | PC4 | Linear Model | 0.276 | 0.0014 |
| Pronotum width | PC2 | Linear Model | 0.662 | 0.0000 |
| Elytra length | PC1 | Linear Model | 0.840 | 0.0000 |
| Abdomen width | PC2 | Linear Model | 0.537 | 0.0000 |
| Body length | PC3 | Linear Model | 1.000 | 0.0000 |

**FIGURE S1**. Correlation between Traits and Axes (HL: head length; HW: head width; AL: antenna length; PL: pronotum length; PW: pronotum width; EL: elytra length; AW: abdomen width; BL: body length)

**TABLE S7.** Tree composition

|  | Mt.Mazongling | | | | | | | Mt.Tiantangzhai | | | | | | | | | |
| --- | --- | --- | --- | --- | --- | --- | --- | --- | --- | --- | --- | --- | --- | --- | --- | --- | --- |
|  | 1 | 2 | 3 | 4 | 5 | 6 | 7 | 1 | 2 | 3 | 4 | 5 | 6 | 7 | 8 | 9 | 10 |
| *Acer davidii* | 1 |  |  |  |  |  |  |  | 1 |  |  |  |  |  | 1 | 1 |  |
| *Acer davidii subsp. grosseri* |  |  |  |  |  |  |  |  |  | 1 |  |  |  |  |  |  |  |
| *Acer henryi* |  |  |  |  |  |  | 2 |  |  |  |  |  |  |  |  |  |  |
| *Acer linganense* |  |  |  |  |  |  |  |  |  | 1 |  |  |  |  |  |  |  |
| *Acer miyabei* | 2 | 2 |  |  |  |  | 3 |  |  |  |  |  |  |  |  |  |  |
| *Acer nikoense* |  |  |  |  |  |  |  |  |  |  |  |  |  |  | 1 |  |  |
| *Acer plmatum* |  |  | 1 | 2 |  |  |  |  |  | 1 |  |  |  |  |  |  |  |
| *Ailanthus altissima* |  |  |  |  |  |  |  |  |  | 1 |  |  | 1 |  |  |  |  |
| *Alangium chinense* |  |  |  |  | 1 |  | 3 |  |  |  |  |  |  |  |  |  |  |
| *Albizia julibrissin* | 1 |  |  |  |  | 1 |  |  |  |  |  |  |  |  |  |  |  |
| *Albizia kalkora* |  |  |  | 1 |  |  | 1 | 2 |  |  |  |  |  |  |  |  |  |
| *Camellia oleifera* |  |  |  |  |  | 1 |  | 12 |  |  |  |  |  |  |  |  |  |
| *Carpinus cordata* |  |  |  |  |  |  |  |  |  | 2 |  |  |  |  |  |  |  |
| *Carpinus turczaninowii* | 2 |  |  | 1 | 2 |  |  |  | 1 | 2 |  |  | 1 | 2 |  |  |  |
| *Carpinus viminea* |  |  |  |  |  |  |  |  |  | 1 | 2 |  |  |  |  |  |  |
| *Castanea seguinii* | 1 | 2 | 3 | 1 | 2 | 1 |  |  |  | 4 | 3 | 1 |  |  | 5 | 6 |  |
| *Celtis bungeana* | 2 |  |  |  |  |  |  |  |  |  |  |  |  |  |  |  |  |
| *Cladrastis wilsonii* |  |  |  |  |  |  |  |  |  |  |  | 2 |  |  |  |  |  |
| *Cornus controversa Hemsl.* | 1 | 2 |  |  | 4 | 1 | 13 | 1 |  |  | 1 | 1 | 1 |  |  |  | 3 |
| *Cornus kousa subsp. chinensis* |  |  |  |  | 2 |  |  |  |  | 1 |  | 2 | 5 |  |  |  |  |
| *Corylopsis sinensis* |  |  |  |  |  |  |  |  |  | 5 |  | 2 |  |  |  |  |  |
| *Cunninghamia lanceolata* |  | 31 | 2 |  |  |  | 9 |  |  |  |  |  |  |  |  |  |  |
| *Cyclocarya paliurus* | 1 | 1 |  |  |  |  |  |  |  |  |  |  |  |  |  |  |  |
| *Dalbergia hupeana* | 6 |  | 1 |  |  |  |  | 19 |  |  |  |  |  |  |  |  |  |
| *Daphniphyllum macropodum* |  |  |  |  |  |  |  |  |  |  |  | 1 |  |  |  |  |  |
| *Diospyros lotus* | 2 | 5 |  |  | 1 | 1 | 3 |  |  |  |  |  | 1 |  |  |  |  |
| *Emmenopterys henryi* |  |  |  |  |  |  |  |  |  |  |  | 1 |  |  |  |  |  |
| *Euscaphis japonica* |  |  |  | 1 |  | 2 |  |  |  |  |  |  |  |  |  |  |  |
| *Evodia fargesii* |  |  |  |  | 2 |  |  |  |  |  |  |  |  |  |  |  |  |
| *Fraxinus chinensis* |  |  | 1 |  |  |  |  |  |  |  |  |  |  |  |  |  |  |
| *Hamamelis mollis* |  |  |  |  |  |  |  |  |  |  |  |  |  | 1 |  |  |  |
| *Hovenia acerba* | 2 |  |  |  |  |  |  |  |  |  |  |  |  |  |  |  |  |
| *Idesia polycarpa* |  |  |  |  | 1 |  |  |  |  |  |  | 3 |  |  |  |  |  |
| *Ilex macropoda* |  |  |  |  |  |  |  |  |  | 3 |  | 1 |  | 8 |  | 1 |  |
| *Juglans cathayensis Dode var. formosana* |  | 7 |  |  |  |  |  |  |  |  |  |  |  |  |  |  |  |
| *Kalopanax septemlobus* |  |  |  |  |  |  | 1 |  |  |  |  |  |  |  |  |  |  |
| *Lindera erythrocarpa* | 2 | 1 |  |  | 1 | 4 |  | 2 |  |  |  |  |  | 1 | 1 | 5 |  |
| *Lindera glauca* | 1 |  | 1 | 9 |  | 2 |  | 1 |  |  | 9 |  | 3 | 2 |  |  |  |
| *Lindera obtusiloba* |  |  |  |  |  |  | 3 |  |  |  |  |  |  |  | 6 | 2 |  |
| *Liquidambar formosana* |  |  |  | 1 |  |  |  | 9 | 19 |  |  |  |  |  |  |  |  |
| *Litsea coreana Levl. var. sinensis* | 11 |  |  |  |  |  |  |  |  |  |  |  |  |  |  |  |  |
| *Litsea elongata* |  |  |  |  |  |  |  |  |  |  | 4 | 7 |  |  |  |  |  |
| *Maackia hupehensis* |  |  |  |  |  |  |  |  |  | 1 |  |  |  |  |  |  |  |
| *Mallotus tenuifolius* |  | 1 |  |  |  |  |  |  |  |  |  |  |  |  |  |  |  |
| *Malus hupehensis* |  |  |  |  |  |  |  |  |  |  |  |  |  |  | 1 |  |  |
| *Meliosma oldhamii* |  |  |  |  |  |  |  |  |  |  | 3 |  |  |  | 4 |  |  |
| *Meliosma pinnata* |  |  |  |  |  |  |  |  |  |  | 1 |  |  |  |  |  |  |
| *Meliosma veitchiorum* |  |  |  |  |  |  |  |  |  |  |  | 1 | 1 |  |  | 1 |  |
| *Morus alba* |  |  |  |  |  |  | 1 |  |  |  |  |  |  |  |  |  |  |
| *Photinia beauverdiana* |  |  |  |  |  | 1 |  |  |  |  |  |  | 2 |  |  |  |  |
| *Photinia hirsuta* |  |  |  |  |  |  |  |  |  |  |  |  |  | 1 |  |  |  |
| *Photinia megaphylla* |  |  |  |  |  |  |  |  |  | 1 |  |  |  |  |  |  |  |
| *Photinia parvifolia* |  |  |  |  |  |  |  |  |  | 1 |  |  |  |  |  |  |  |
| *Pinus hwangshanensis* |  |  | 14 |  |  | 1 | 1 |  |  | 1 |  |  | 2 | 1 | 5 | 31 | 58 |
| *Platycarya strobilacea* |  | 1 | 2 | 3 | 3 | 3 | 1 | 1 |  | 7 | 7 |  | 3 |  | 1 |  |  |
| *Prunus dielsiana* |  |  |  |  |  |  |  | 1 |  |  |  |  |  |  |  | 5 |  |
| *Prunus padus* |  |  | 2 |  |  |  |  |  |  |  |  |  |  |  |  |  |  |
| *Prunus spp.* |  |  | 1 |  | 2 |  |  | 2 |  | 3 |  |  | 1 | 3 | 2 |  |  |
| *Pterocarya stenoptera* |  |  |  |  |  |  |  |  | 1 |  |  |  |  |  |  |  |  |
| *Pterostyrax corymbosus* |  |  | 1 |  | 2 |  |  |  |  |  | 2 |  |  |  | 3 |  |  |
| *Quercus aliena var. acutiserrata* |  |  |  |  |  |  |  |  |  |  |  |  |  | 3 | 10 |  | 2 |
| *Quercus glandulifera var. brevipetiolata* |  |  |  | 1 | 3 | 5 |  |  |  |  |  |  |  |  |  |  |  |
| *Quercus glauca* | 2 |  |  |  |  |  |  | 4 | 2 |  |  |  |  |  |  |  |  |
| *Quercus myrsinifolia* |  |  |  |  |  |  |  |  |  | 21 | 9 | 14 | 4 |  |  |  |  |
| *Quercus serrata* |  |  |  |  |  |  |  |  |  | 1 | 1 |  | 6 | 13 |  | 1 |  |
| *Quercus variabilis* | 2 |  |  |  |  |  |  |  |  |  |  |  |  |  |  |  |  |
| *Rhus chinensis* |  |  |  |  | 1 | 1 |  |  |  |  |  |  |  |  |  |  |  |
| *Sorbus alnifolia* |  |  |  |  |  |  |  |  |  |  |  | 1 |  | 1 |  | 1 | 1 |
| *Sorbus folgneri* |  |  |  |  |  |  |  |  |  |  | 1 |  |  |  |  |  |  |
| *Stewartia sinensis* |  |  |  |  |  |  |  |  |  |  |  | 1 | 7 | 7 |  |  |  |
| *Styrax japonicus* |  |  |  |  |  |  |  |  |  |  |  |  |  |  | 12 |  |  |
| *Styrax obassia* |  |  |  |  |  | 1 |  |  |  |  |  |  | 1 |  |  |  |  |
| *Symplocos tanakana* |  |  |  |  |  | 1 | 1 | 2 |  |  | 1 | 3 | 2 | 16 | 1 |  | 4 |
| *Tetradium glabrifolium* |  |  |  |  |  |  |  |  |  | 1 |  |  |  |  |  |  |  |
| *Tilia japonica* | 2 |  |  |  |  |  |  |  |  |  |  |  | 1 |  |  |  |  |
| *Toxicodendron succedaneum* |  |  |  |  |  |  |  | 1 |  | 1 |  |  |  |  |  |  |  |
| *Viburnum sympodiale* |  |  |  |  |  |  |  |  |  |  |  | 2 |  |  |  |  |  |
| *Yulania denudata* |  |  |  |  |  |  |  |  |  |  |  | 1 |  |  |  |  |  |

**TABLE S8.** Data of environmental factors

| Environmental factors | Mean_SD | Range |
| --- | --- | --- |
| Elevation | 1053.94 ± 296.48 | 630-1587 |
| NDVI | 0.51 ± 0.05 | 0.398-0.561 |
| Canopy cover | 0.84 ± 0.06 | 0.70-0.90 |
| DBH | 13.67 ± 3.12 | 8.65-18.17 |
| Air temperature | 19.12 ± 1.56 | 16.40-21.80 |
| Air humidity | 90.22 ± 2.78 | 86.2-95.06 |
| Slope | 23.06 ± 9.39 | 2-39 |
| Litter depth | 2.19 ± 0.49 | 1.4-3.2 |


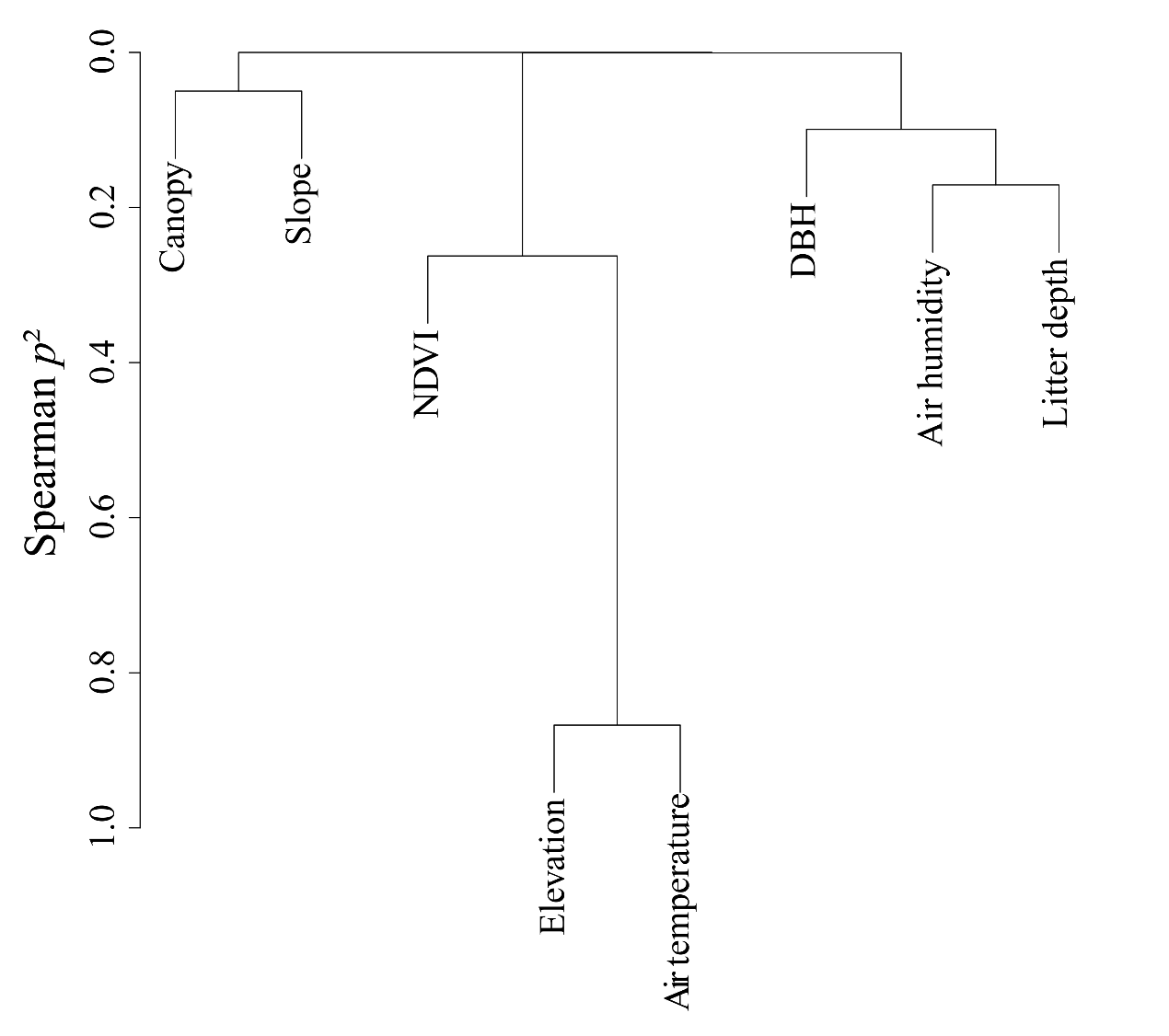


**FIGURE S2.** The correlation of environmental factors

**REFERENCES**

Barton, P. S., Gibb, H., Manning, A. D., Lindenmayer, D. B., & Cunningham, S. A. (2011). Morphological traits as predictors of diet and microhabitat use in a diverse beetle assemblage: MORPHOLOGICAL TRAITS OF BEETLES. *Biological Journal of the Linnean Society*, *102*(2), 301–310. https://doi.org/10.1111/j.1095-8312.2010.01580.x

Fountain‐Jones, N. M., Baker, S. C., & Jordan, G. J. (2015). Moving beyond the guild concept: Developing a practical functional trait framework for terrestrial beetles. *Ecological Entomology*, *40*(1), 1–13. https://doi.org/10.1111/een.12158

Fountain-Jones, N. M., Jordan, G. J., Burridge, C., Wardlaw, T. J., Baker, T. P., Forster, L., & Baker, S. C. (2017). *Trophic position determines functional and phylogenetic recovery after disturbance within a community*.

Gillespie, M. A. K., Birkemoe, T., & Sverdrup‐Thygeson, A. (2017). Interactions between body size, abundance, seasonality, and phenology in forest beetles. *Ecology and Evolution*, *7*(4), 1091–1100. https://doi.org/10.1002/ece3.2732

Hagge, J., Müller, J., Birkemoe, T., Buse, J., Christensen, R. H. B., Gossner, M. M., Gruppe, A., Heibl, C., Jarzabek‐Müller, A., Seibold, S., Siitonen, J., Soutinho, J. G., Sverdrup‐Thygeson, A., Thorn, S., & Drag, L. (2021). What does a threatened saproxylic beetle look like? Modelling extinction risk using a new morphological trait database. *Journal of Animal Ecology*, *90*(8), 1934–1947. https://doi.org/10.1111/1365-2656.13512

Pérez-Sánchez, D., Galante, E., & Micó, E. (2020). Functional and Taxonomic Beta Diversity of Saproxylic Beetles in Mediterranean Forests: On What Factors Do They Depend? *Environmental Entomology*, *49*(3), 615–626. https://doi.org/10.1093/ee/nvaa045

Thompson, R. T. (1992). Observations on the morphology and classification of weevils (Coleoptera, Curculionoidea) with a key to major groups. *Journal of Natural History*, *26*(4), 835–891. https://doi.org/10.1080/00222939200770511

Vandewalle, M., de Bello, F., Berg, M. P., Bolger, T., Dolédec, S., Dubs, F., Feld, C. K., Harrington, R., Harrison, P. A., Lavorel, S., da Silva, P. M., Moretti, M., Niemelä, J., Santos, P., Sattler, T., Sousa, J. P., Sykes, M. T., Vanbergen, A. J., & Woodcock, B. A. (2010). Functional traits as indicators of biodiversity response to land use changes across ecosystems and organisms. *Biodiversity and Conservation*, *19*(10), 2921–2947. https://doi.org/10.1007/s10531-010-9798-9
